# Artificial Intelligence in Head and Neck Cancer: An Umbrella Review

**DOI:** 10.7759/cureus.108154

**Published:** 2026-05-02

**Authors:** George V Joy, Jibin Kunjavara, Kamaruddeen Mannethodi, Amel Daw, Abdulqadir J Nashwan

**Affiliations:** 1 Nursing &amp; Midwifery Research, Hamad Medical Corporation, Doha, QAT

**Keywords:** artificial intelligence (ai), convolutional neural networks (cnn), deep learning (dl), diagnostics, head and neck cancer (hnc), machine learning (ml), prognostics, radiomics, systematic reviews, treatment planning

## Abstract

Head and neck cancers (HNCs) present significant challenges in diagnosis, treatment planning, and prognostication due to their heterogeneous nature and anatomical complexity. Artificial intelligence (AI), particularly convolutional neural networks (CNNs), has emerged as a transformative tool for developing clinical decision support systems (CDSS) that enhance precision in oncology. However, the breadth of AI applications in HNC has often obscured specific insights into their clinical impact.

This umbrella review critically evaluates the role of AI-supported CDSS, with a focus on CNN-based models, in improving diagnostic accuracy, guiding treatment decisions, and refining prognostic predictions in HNC. Systematic reviews (SRs) published on AI applications in HNC were identified and synthesized. Data were extracted on clinical utility, methodological rigor, and reported limitations. The methodological quality of the included reviews was assessed using A Measurement Tool to Assess Systematic Reviews-2 (AMSTAR-2) to ensure reliability of the synthesized evidence. A total of 47 SRs met the inclusion criteria. CNN-driven CDSS demonstrated strong performance in diagnostic imaging and histopathology, with accuracy often comparable to or surpassing that of expert clinicians. In treatment planning, AI-assisted models improved tumor delineation, predicted radiotherapy-related toxicities, and provided intraoperative decision support through modalities such as hyperspectral imaging (HSI) and optical coherence tomography. Prognostic CDSS integrating radiomics, clinical, and molecular data outperformed traditional staging systems in predicting recurrence and survival.

Nevertheless, widespread clinical translation is limited by retrospective study designs, small and heterogeneous datasets, lack of external validation, and concerns about interpretability. This review provides precise insights into how CNN-based models can enhance clinical decision-making in HNC. These systems hold the potential to transform oncology practice by improving diagnostic reliability, optimizing therapeutic strategies, and enabling personalized prognostic assessments. Future research should prioritize prospective multi-center validation, standardized evaluation protocols, and the development of interpretable models to ensure safe and effective integration of AI-CDSS into clinical care.

## Introduction and background

Head and neck cancer (HNC), a multi-faceted and life-altering disease, encompasses malignancies arising from the mucosal linings of the oral cavity, pharynx, larynx, nasal cavity, and paranasal sinuses, presenting a significant global health challenge that demands innovative solutions [1]. Despite advances in medical technology, challenges persist in achieving timely and accurate diagnosis, effective treatment planning, and reliable prognostic predictions. These challenges are often compounded by the anatomical region's complexity and the heterogeneous nature of the disease [2]. The introduction of artificial intelligence (AI) into oncology represents a transformative opportunity to address these issues, leveraging machine learning (ML), deep learning (DL), and neural network (NN) methodologies to enhance the precision and efficiency of cancer care [3].

AI applications in HNC have demonstrated potential across three critical domains: diagnosis, treatment, and prognosis. In diagnosis, AI algorithms, particularly those based on DL, are increasingly utilized for image analysis in radiology and pathology [4]. These algorithms can process vast amounts of imaging data, identifying subtle patterns that may elude human observation, thereby improving diagnostic accuracy and reducing inter-observer variability. Similarly, AI-driven models are aiding in treatment planning, such as radiotherapy optimization, where precision is paramount to minimize damage to surrounding tissues while targeting cancerous cells [5]. Prognostic models powered by AI are also gaining traction, providing clinicians with tools to more accurately predict patient outcomes and tailor treatment accordingly [6].

Systematic reviews (SRs) have become essential for synthesizing evidence on the effectiveness of AI applications in HNC. These reviews aggregate findings from multiple studies, providing a comprehensive understanding of the strengths, limitations, and overall impact of AI in clinical settings [7]. However, the rapid pace of AI development and the variability in study designs, methodologies, and reporting standards pose challenges to drawing consistent conclusions. An umbrella review, which synthesizes evidence from SRs, is therefore well-suited to critically assess the current state of knowledge, methodological quality, and research gaps [8].

This review evaluates the utility of AI, with particular emphasis on clinical decision support systems (CDSS) and convolutional neural networks (CNNs), in enhancing diagnostic accuracy, treatment outcomes, and prognostic prediction for patients with HNC. Through SRs, the study will identify promising AI-driven tools and techniques, assess their performance metrics, and explore their potential integration into clinical workflows. Additionally, it will evaluate the methodological quality of the included reviews using tools such as A Measurement Tool to Assess Systematic Reviews-2 (AMSTAR-2), offering insights into the reliability and robustness of the synthesized evidence [3].

This umbrella review, therefore, aims to comprehensively examine the role of AI-supported CDSS in the management of HNC by synthesizing evidence from existing SRs. It focuses on elucidating the clinical applications of AI, particularly CNN-based models, in supporting diagnostic precision, informing treatment planning, and improving patient outcomes, while also critically appraising the methodological quality and rigor of the included reviews. A key focus of this review is to uncover research gaps and methodological limitations that may hinder the effective implementation of AI-based CDSS in head and neck oncology. Challenges such as the predominant reliance on retrospective datasets, inconsistent algorithm performance due to variable data quality, limited sample sizes, and heterogeneity in patient demographics underscore the urgent need for standardized evaluation protocols and robust validation frameworks. By mapping emerging trends, consolidating current knowledge, and identifying areas in need of further investigation, this review seeks to inform future research directions, support evidence-based clinical practice, and facilitate the meaningful integration of AI into oncological care. Ultimately, it aims to bridge the gap between technological innovation and clinical utility, with the overarching goal of improving patient outcomes in HNC.

## Review

Methods

Eligibility Criteria

The eligibility criteria for this umbrella review are carefully defined to ensure the inclusion of relevant and high-quality studies. SRs that focus on the use of AI in HNC are eligible for inclusion. The population of interest includes patients diagnosed with or at risk for HNC. AI techniques, such as ML, DL, and NNs, must be central to the study, with outcomes examining diagnostic accuracy, treatment efficacy, prognostic performance, or cost-effectiveness. The search has included SRs published until July 2025, enabling a comprehensive evaluation of the evidence. Importantly, only studies available in full text are included to ensure thorough analysis. Exclusion criteria include reviews not focused on AI or HNC, studies without systematic methods, and publications in non-English languages if translation resources are unavailable.

Information Sources

A systematic search was conducted across five major databases: PubMed, EMBASE, Cochrane Library, Web of Science, and Scopus. Keywords and phrases, including "artificial intelligence," "machine learning," "deep learning," "neural networks," and "head and neck cancer," were combined with Boolean operators to refine the search results. For example, the search string incorporated terms such as ("artificial intelligence" OR "machine learning") AND ("head and neck cancer") AND ("systematic review" OR "meta-analysis"). This approach ensured that all potentially relevant studies were identified and considered for inclusion. The selection process for eligible studies was documented in accordance with the Preferred Reporting Items for Systematic Reviews and Meta-analyses (PRISMA) guidelines.

Data Management

Data extraction was conducted systematically using a structured template created in Microsoft Excel. Extracted data included details such as author(s), publication year, journal, study objectives, AI tools or techniques employed, performance metrics, and outcomes of interest. The outcomes focused on measures of diagnostic accuracy (e.g., sensitivity, specificity), treatment efficacy (e.g., odds ratios and relative risks), and prognostic performance. Two independent reviewers performed the data extraction to ensure accuracy and consistency. Any discrepancies were resolved through discussion or, when necessary, by consulting a third reviewer. This approach minimized bias and enhanced the reliability of the findings.

Selection Process

The selection process was carried out in two main stages: title and abstract screening, followed by full-text review. References retrieved from the database searches were initially imported into Zotero for organization, and duplicate entries were removed. During the screening phase, two independent reviewers (AD, GJ) assessed titles and abstracts against the predefined inclusion and exclusion criteria. Articles deemed potentially relevant proceeded to full-text review, during which the eligibility of each study was confirmed. Disagreements at any stage were resolved through consensus or, when necessary, adjudication by a third reviewer. This rigorous selection process ensured that only high-quality and relevant studies were included in the review.

Data Synthesis

Data synthesis was conducted using a structured approach. A data extraction template was created in Microsoft Excel to systematically collect relevant information. Three independent reviewers (GJ, KM, JK) extracted the data to ensure accuracy and minimize bias, with any discrepancies resolved through discussion or consultation with a fourth reviewer (AN). Extracted data included study details (e.g., authors, publication year, journal, objectives, and methods), AI-related characteristics (e.g., algorithms used, performance metrics), and outcomes (e.g., diagnostic accuracy, treatment effectiveness, and prognostic insights). For studies derived from the same dataset, individual results were extracted and aggregated as appropriate to avoid duplication and ensure a comprehensive synthesis.

Methodological Quality

The methodological quality of the included SRs was evaluated using quality assessment tools, such as AMSTAR-2. Although this review focused on identifying and narratively describing the quality checklists used in each study, no exclusions were made based on the presence or absence of a formal quality appraisal. Each SR that reported quality assessment was examined against these criteria, and the reflections on methodological quality were synthesized narratively in the results section.

Data Analysis

**Figure 1:**
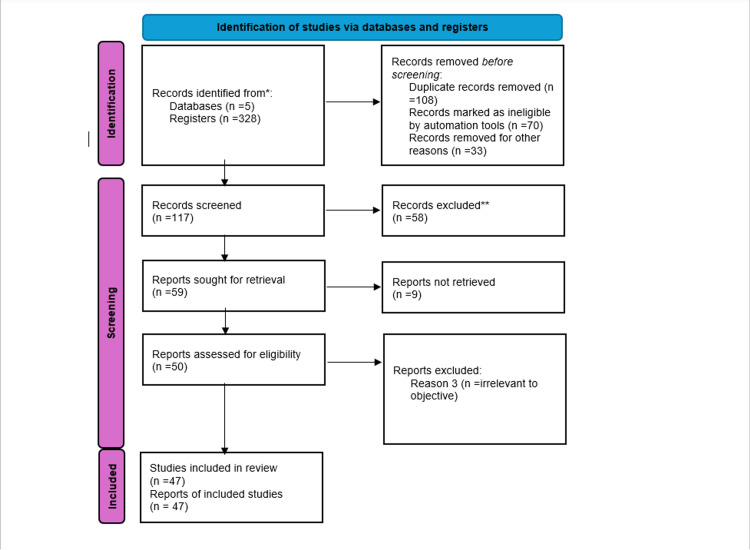
The PRISMA flow diagram PRISMA: Preferred Reporting Items for Systematic Reviews and Meta-analyses

The data were analyzed qualitatively through thematic synthesis to identify prevailing trends, research gaps, and emerging themes in the application of AI in HNCs. For studies reporting quantitative outcomes, a summary table was developed to present key findings, emphasizing performance metrics and clinical outcomes. A meta-analysis (MA) was not conducted due to significant heterogeneity in study designs, AI methodologies, outcome measures, and reporting standards across the included reviews. This variation precluded the meaningful aggregation of effect sizes and statistical comparison. Instead, the synthesis focused on extracting and organizing salient themes to provide a narrative understanding of the current landscape. This approach enabled a comprehensive overview of AI applications in HNC, particularly regarding diagnostic accuracy, treatment effectiveness, prognostic modeling, and directions for future research. To ensure transparency in the study selection process, the PRISMA 2020 flow diagram was used to illustrate the number of records identified, screened, assessed for eligibility, and included in the final synthesis (Figure 1). 

Results

This umbrella review included 47 SRs evaluating the application of AI in the diagnosis, treatment, and prognosis of HNC, including specific subtypes such as thyroid, oral, and nasopharyngeal cancers. Collectively, these studies incorporated a broad spectrum of AI methodologies, ranging from conventional ML algorithms to advanced DL architectures, including CNNs, support vector machines (SVMs), and radiomics-enhanced models. The sample sizes across the included reviews varied substantially, with some synthesizing data from as few as seven studies and others encompassing over 100 primary articles. Table 1 summarizes studies on AI applications in cancer diagnosis and management, outlining key findings, limitations, and future directions.

**Table 1:** Studies on AI applications in HNCs diagnosis and management AI: Artificial intelligence; ML: Machine learning; DL: Deep learning; SR: Systematic review; MA: Meta-analysis; CAD: Computer-aided diagnosis; CNN: Convolutional neural network; SVM: Support vector machine; LDA: Linear discriminant analysis; NN: Neural network; PET: Positron emission tomography; VS: Vestibular schwannoma; LN: Lymph node; PRISMA: Preferred Reporting Items for Systematic Reviews and Meta-analyses; NIH: National Institutes of Health; OPMD: Oral potentially malignant disorders; US: Ultrasound; HSI: Hyperspectral imaging; NPC: Nasopharyngeal carcinoma; AUC: Area under the curve; DOR: Diagnostic odds ratio; ANN: Artificial neural network; TI-RADS: Thyroid Imaging Reporting and Data System; TRIPOD: Transparent Reporting of a Multivariable Prediction Model for Individual Prognosis or Diagnosis; PROBAST: Prediction Model Risk of Bias Assessment Tool; STARD: Standards for Reporting Diagnostic Accuracy Studies

Study	Study Type	Number of Studies	Cancer Type	AI Technique Used-	Primary Objective	Key Findings	Challenges /Limitations	Clinical Implications & Future Directions
Lima et al. [9]	SR & MA	6	Retinoblastoma	Supervised/semi-supervised ML	To investigate the pooled performance of AI methods for diagnosing retinoblastoma based on fundus images	Sensitivity 98.2%, Specificity 98.5%, AUC 0.986	Heterogeneity, lack of uniform computational power comparison, and little population data	High diagnostic performance for retinoblastoma detection, useful for high-risk populations. Prospective validation and cost-effectiveness studies in varied income settings.
Mahmood et al. [10]	SR	11	HNC	Combination of heuristics + supervised and unsupervised ML	To assess AI/ML accuracy for the detection & grading of potentially malignant/cancerous head & neck lesions	Accuracy between 79-100%	Limited evidence for other head & neck lesions beyond OPMD	Early evidence of ML as a diagnostic aid for some OPMD lesions. Development of state-of-the-art DL techniques.
Nernekli et al. [11]	SR	12	Vestibular	U-Net + Convolutional-Attention Transformer	Review of data sources, algorithms & metrics in VS segmentation	Automatic segmentation offers precision & efficiency	Domain shift across datasets, need for domain adaptation	Could improve tumor growth tracking & clinical decision-making. Develop robust models, address domain shift, and enable secure data sharing.
He et al. [12]	SR & MA	23	HNC	Bayesian network MA	To assess relative efficacy & safety of second-line treatments for platinum-resistant recurrent/metastatic head and neck squamous cell carcinoma	PD-1 inhibitors improved overall survival, objective response rate, and treatment-related adverse event outcomes compared with the standard of care. Afatinib showed better progression-free survival and objective response rate than the standard of care.	Indirect comparisons, small phase II trials, limited subgroup analysis, and generalizability	PD-1 inhibitors improve overall survival, afatinib improves progression-free survival; PX-866 and others show no overall survival/progression-free survival benefit.
Xu et al. [13]	SR & MA	19	Thyroid	CAD systems (ML & DL based)	Determine the accuracy of CAD systems in diagnosing malignant thyroid nodules	ML-based: Sensitivity 0.86, Specificity 0.85; DL-based: Sensitivity 0.89, Specificity 0.84.	Statistical heterogeneity, different reference standards, and potential influence from ultrasonic diagnosis	Both ML & DL CAD systems show good diagnostic performance for thyroid nodules. Further studies on standardization and system optimization.
Santer et al. [14]	SR	13	Head and neck squamous cell carcinoma	AI-radio imaging	SR of AI’s role in classifying LNs in locally-advanced head and neck squamous cell carcinoma following PRISMA and NIH Study Quality Assessment Tool	AI is a promising diagnostic tool for LN-classification in head and neck squamous cell carcinoma, with diagnostic accuracy >85%, reaching up to 99% in some studies.	Retrospective, mostly single-center studies; limited focus on laryngeal, nasopharyngeal, or salivary gland cancers; primarily used CT and PET-CT; no studies used MRI for AI-based classification	High diagnostic potential for AI in LN classification; could improve diagnostic support in clinical practice. Need for well-powered, prospective RCTs to further validate AI’s role in LN-classification in advanced head and neck squamous cell carcinoma.
Loperfido et al. [15]	SR	12	HNC	AI, ML	Describe AI’s applications in head and neck oncology, focusing on surgical use	Focus areas: AI for surgical margin assessment (7 articles), complications assessment (4 papers), salvage laryngectomy indication (1 paper).	Limited number of studies; applications still narrow; practical role of AI in surgery yet to be clearly defined	AI is emerging as a valuable tool in surgical oncology, but clinicians must retain critical decision-making roles Continuous need for clinicians to critically evaluate AI outputs and integrate with their clinical judgment; further research is needed.
Adeoye et al. [16]	SR	53	HNC	Data-centric AI	Overview of data quality issues in datasets used for ML models in HNC	Data quality was rarely assessed prior to ML model development, regardless of dataset type.	Exclusion of some large public datasets to avoid bias; metadata used for data quality checks; models focused mainly on classification tasks	Studies should implement standardized data quality assessments and adhere to TRIPOD/PROBAST/STARD guidelines to improve model reliability. Introduce robust data quality evaluations and continuous model updates during ML model development to enhance generalizability in HNC AI applications.
Pirayesh et al. [17]	SR & MA	7	Oral cell carcinoma	DL	SR and MA on DL models for oral squamous cell carcinoma detection using histopathology images	DL models demonstrated high accuracy: segmentation (accuracy 0.97, sensitivity 0.97, specificity 0.98); classification (accuracy 0.99, sensitivity 0.99, specificity 1.0); MA pooled sensitivity 0.98, specificity 0.93.	Language bias due to English-only studies; limited head-to-head model comparisons; single-pathologist reference standards; evolving DL methods may make findings outdated	DL approaches show strong potential for automated OSCC diagnosis, with performance comparable to that of human experts. Conduct further DL studies following optimal study designs to reduce bias and enhance clinical utility; improve generalizability and robustness of DL models for histopathological diagnosis.
Li et al. [18]	SR & MA	17	Oral cell carcinoma	ML algorithms	Comprehensive overview of AI’s application in detecting OPMD and oral cancerous lesions	AI-assisted screening yielded high diagnostic performance: sensitivity 89.9%, specificity 89.2%, DOR 68.4, AUC 0.938.	Language bias from including only English studies; heterogeneity due to diverse imaging modalities used across studies	AI can assist in improving early detection and screening, especially in low- and middle-income countries with limited access to specialists. Expect significant improvements with evolving imaging technologies and AI algorithms; potential to enhance healthcare outcomes by enabling large-scale, accurate screening.
Alabi et al. [19]	SR	45	HNC	AI + Radiomics (ML/DL)	Examine AI-based radiomics applications in managing HNC	AI-based radiomics aids in tumor staging, grading, malignancy classification, and prognosis.	Data imbalance, feature reproducibility, lack of external validation, and modality variations	AI-radiomics enhances diagnostic evaluation and decision-making for HNC. Explore multi-modal fusion to improve model performance.
Potipimpanon et al. [20]	SR & MA	25	Thyroid nodules	AI	Assess the pooled sensitivity and specificity of AI vs radiologists in thyroid US imaging	AI sensitivity/specificity: 0.86/0.78; Radiologist: 0.85/0.82; AUC comparable (AI: 0.89, Radiologist: 0.91).	Heterogeneity due to retrospective designs and Asian-dominant datasets limiting generalizability	AI performs comparably to radiologists and can be a clinical support tool. Further studies to validate AI’s equivalence in broader populations.
Kavyashree et al. [21]	SR	73	Oral cancer	AI/ML/DL	Overview of AI techniques to automate and improve oral cancer detection	AI techniques show >90% accuracy in most models.	Limited datasets, misclassification, patch detection issues, need for multi-modal solutions, and real-time diagnosis limitations	AI reduces diagnostic time, supporting faster clinical decision-making. Larger datasets, enhanced algorithms, and real-time model development are needed.
Xue et al. [22]	SR & MA	25	Thyroid nodules	AI (CAD, DL, ML)	Clarify AI accuracy in differentiating benign/malignant thyroid nodules via US	Sensitivity: 0.88, Specificity: 0.81, AUC: 0.92; AI performs better in populations <50 years.	Statistical heterogeneity due to AI model diversity, incomplete TI-RADS data	AI improves diagnostic efficacy for thyroid nodules, especially in younger populations. Further refinement of AI techniques and larger subgroup analyses are needed.
Wu et al. [23]	SR	7	HNC	CNN, SVM, LDA, CAD	Review recent advances in HSI + CAD for HNC detection	LDA showed highest accuracy (92%) vs CNN (82%) for HNC detection.	Sensitivity to external conditions, high data requirements, lack of spectral libraries	HSI shows promise for multi-scale tissue diagnostics in HNC. Develop spectral libraries, integrate dimensionality reduction, and hybrid ML models.
Khanagar et al. [24]	SR	19	Oral cancer	ML, DL, AI	To assess the utilization of AI in the diagnosis, classification, and prediction of oral cancer using histopathological images	Accuracy ranged 89.47%-100%, sensitivity 97.76%-99.26%, specificity 92%-99.42%. Outperformed clinical methods.	Interpretability and explainability of AI models are lacking; no unified method for interpretability	DL has enhanced oncology by assisting pathologists and improving disease management. Promote interdisciplinary AI integration; future research should focus on improving the explainability of AI models.
Ng et al. [25]	SR	60	Nasophrangeal	AI, ML, DL	Explore clinical application and direction of AI in NPC management	AI applications included auto-contouring, diagnosis, prognosis, and radiotherapy planning. CNNs were the most used DL models, and ANN for ML.	Limited generalizability due to single-institution datasets and small sample sizes (33% had ≤150 samples)	AI shows promise in prognostic, diagnostic, and auto-contouring roles, offering individualized treatment plans. Encourage multi-center studies with larger datasets; external validation needed.
Beristain-Colorado et al. [26]	SR	9	Oral cancer	AI, NNs	To assess evidence on the sensitivity and specificity of NNs for oral cancer detection	Most studies reported accuracy >85%.	Limited sample sizes, some studies tested on the same dataset used for training	CNN-based models support early cancer detection and can aid remote clinicians. Combine AI tools with patient-specific risk factors for improved diagnostics.
Bassani et al. [2]	SR	13	HNC	AI, ML	To evaluate AI evolution in diagnosing head and neck neoplasms	Accuracy >90% across studies, high AUC, sensitivity, and specificity.	Salivary gland tumors present a diagnostic challenge due to morphologic overlap	AI improves diagnostic capacity for HNC, especially in cytology. Expand AI to non-SCC tumors like salivary gland neoplasms; larger datasets and real-world integration are recommended.
Alabi et al. [27]	SR	41	Oral squamous cell carcinoma	ML	Assess the role of ML in oral squamous cell carcinoma management	ML models demonstrated high accuracy in detecting oral squamous cell carcinoma, but clinical implementation remains low due to limited validation.	Small datasets, high variability in ML models, lack of prospective validation	ML can improve early detection and personalized treatment. Large-scale validation studies and clinical trials are needed.
Gouthamchand et al. [28]	SR	23	Head and neck squamous cell carcinoma	Handcrafted radiomics vs. DL	Compare handcrafted radiomics and DL for prognosis	DL models slightly outperformed handcrafted radiomics in most cases, particularly in survival prediction.	Study heterogeneity, lack of external validation	DL could improve patient-specific prognostication. Better generalization of DL models with diverse datasets.
Bisdas et al. [29]	SR & MA	7	HNC	ML-augmented radiomics	Assess ML-augmented radiomics in histopathological diagnosis	ML significantly improved histopathological classification accuracy.	Limited access to raw data, potential publication bias	AI could assist pathologists in cancer diagnosis. More robust studies needed for clinical adoption.
Adeoye et al. [30]	SR	27	Oral cavity cancer	ML	Predict outcomes in oral cavity cancer	ML models accurately predicted recurrence and survival in multiple studies.	High risk of bias, no external validation	Could aid oncologists in risk stratification. Standardized reporting and multi-center validation needed.
Moharrami et al. [31]	SR	34	HNC	ML with structured data	Predict post-treatment outcomes	ML models showed potential in predicting survival and treatment response.	Lack of large, diverse datasets	ML could support post-treatment monitoring. Stronger external validation needed.
Messineo et al. [32]	SR	9	Head and neck squamous cell carcinoma	Radiomics	Evaluate radiomic applications in head and neck squamous cell carcinoma	Radiomics helped in tumor characterization, prognosis, and treatment response.	Variability in radiomic methods, lack of standardization	Potential for non-invasive treatment monitoring. Standardization and large-scale validation are needed.
Giraud et al. [33]	SR	8	HNC	Radiomics & ML	Radiomics and ML in radiotherapy	ML enhanced dose planning and toxicity prediction.	Data variability, integration issues	ML can personalize radiotherapy treatment plans. Clinical validation and prospective trials are required.
Jiang et al. [34]	SR	10	Oral cancer	Radiomics	Predict occult LN metastases	Radiomics improved prediction accuracy of occult metastases compared to clinical models.	Small datasets, no external validation	Could improve surgical planning. Need for standardized feature extraction.
Giannitto et al. [35]	SR	7	HNC	Radiomics-based ML	ML for diagnosing LN metastases	ML achieved high sensitivity and specificity for detecting nodal metastasis.	Variability in imaging protocols	May reduce unnecessary biopsies. Standardized imaging protocols needed.
Wang et al. [36]	SR & MA	24	HNC	PET/CT-based radiomics	Prognostic value of PET/CT radiomics	PET/CT radiomics was significantly associated with patient survival.	Need for harmonized radiomic feature extraction	Useful for risk stratification. Harmonization of radiomics features required.
Michelutti et al. [4]	SR	19	HNC	AI algorithms	Prognosis & detection of LN involvement	AI showed high accuracy in detecting nodal involvement.	Data heterogeneity, need for validation	Could enable personalized treatment strategies. Emphasis on clinical integration.
Devault-Tousignant et al. [37]	SR	5	HNC	AI in reconstructive surgery	Evaluate AI for surgical planning & outcomes	AI improved surgical planning and post-operative monitoring.	Lack of real-world evidence	Could enhance surgical decision-making. Need for real-world clinical trials.
Araújo et al. [5]	SR	28	HNC	ML	Predict toxicities from cancer treatment	ML predicts treatment toxicities with 82% accuracy.	Heterogeneity in ML methodologies across studies	ML can improve pre-treatment risk assessments. Standardizing ML-based toxicity prediction models.
Carbonara et al. [38]	SR	24	HNC	Radiomics & ML	Analyze radiation-induced toxicity risks	Radiomics effectively predicts toxicity risks in radiotherapy patients.	Need for standardization in radiomics toxicity prediction	Radiomics could personalize radiotherapy planning. Developing robust radiomics toxicity prediction algorithms.
Chiesa-Estomba et al. [39]	SR	30	Oral cancer	ML	AI for oral cancer prognosis	AI enhances oral cancer prognosis and clinical decision-making.	AI models require larger clinical validation	AI could support clinical decision-making in oral cancer. Validating AI models in real-world oral cancer settings.
Du et al. [40]	SR	18	Laryngeal cancer	DL	Evaluate DL in laryngoscopy	DL improves laryngoscopy accuracy for tumor detection.	DL in laryngoscopy needs multi-center validation	DL could enhance laryngoscopic tumor detection. Multi-center validation of DL laryngoscopy.
Franzese et al. [6]	SR	21	HNC	AI in radiotherapy workflow	AI in radiotherapy workflow improvement	AI-driven radiotherapy optimizes treatment workflows.	AI integration into radiotherapy faces implementation barriers	AI in radiotherapy may reduce treatment complications. Refining AI-based radiotherapy planning tools.
Jerjes et al. [41]	SR	26	Oral cancer	Optical coherence tomography & AI	AI-assisted oral cancer detection	AI-assisted optical coherence tomography improves oral cancer detection rates.	AI-optical coherence tomography standardization challenges exist	AI-optical coherence tomography integration could refine early oral cancer screening. Establishing clinical guidelines for AI-optical coherence tomography integration.
Jethanandani et al. [42]	SR	22	HNC	Radiomics in MRI	MRI-based radiomics for HNC prognosis	MRI-based radiomics enhances HNC prognosis.	MRI radiomics models require larger datasets	MRI radiomics could optimize HNC staging. Expanding datasets for MRI-based radiomics research.
Kim et al. [43]	SR	25	Oral cancer	Optical coherence tomography	Evaluate optical coherence tomography in oral cancer diagnosis	Optical coherence tomography provides high sensitivity in early oral cancer detection.	Optical coherence tomography lacks real-world clinical application data	Optical coherence tomography may serve as a non-invasive biopsy alternative. Conducting longitudinal studies on optical coherence tomography effectiveness.
Kim et al. [44]	SR	23	Oral cancer	AI-assisted image analysis	AI in oral cancer image classification	AI achieves 89% accuracy in oral mucosa cancer classification.	AI in oral mucosa imaging lacks external validation	AI may improve oral cancer screening programs. Deploying AI-based oral cancer detection in hospitals.
Malhotra et al. [45]	SR	29	Oral squamous cell carcinoma	AI vs. biopsy	AI vs biopsy in oral squamous cell carcinoma detection	AI-based oral squamous cell carcinoma detection matches biopsy accuracy.	AI-biopsy comparisons require prospective trials	AI could streamline oral squamous cell carcinoma diagnosis, reducing biopsy reliance. Prospective trials for AI vs biopsy comparisons.
Patil et al. [46]	SR	27	HNC	Genomic ML	Genomic ML in HNC	Genomic ML improves subtype classification in HNC.	Genomic ML requires validation with real-world datasets	Genomic ML may guide precision medicine strategies. Enhancing genomic ML with multi-omics data.
Sant et al. [47]	SR	20	Thyroid cancer	AI for thyroid diagnostics	AI in thyroid nodule diagnostics	AI reduces unnecessary biopsies in thyroid diagnostics.	AI in thyroid nodules needs regulatory approvals	AI could optimize thyroid cancer diagnostics. Securing regulatory approval for AI-based thyroid analysis.
Valizadeh et al. [48]	SR	31	HNC	Radiomics & AI for metastasis detection	AI in diagnosing LN metastases	AI enhances metastasis detection in HNC.	Radiomics models lack cross-center standardization	AI may help non-invasively identify LN metastases. Standardizing radiomics feature extraction protocols.
Volpe et al. [49]	SR	19	HNC	AI for radiation oncology	AI applications in radiation oncology	AI improves radiation oncology decision-making.	AI-based radiation oncology faces real-time clinical adoption barriers	AI-assisted radiation therapy may enhance treatment response. Refining AI-assisted radiation oncology decision tools.
Windisch et al. [50]	SR	15	Central nervous system tumors	ML for tumor segmentation	ML in benign tumor segmentation	ML models accurately segment benign central nervous system tumors.	Central nervous system tumor segmentation ML models require further training	ML-based segmentation could aid in neurosurgical planning. Advancing ML models for CNS tumor segmentation.
Xiao et al. [51]	SR	17	Head and neck squamous carcinoma	AI & long non-coding RNAs	Non-coding RNAs in head and neck squamous carcinoma prognosis	AI predicts non-coding RNA impacts in head and neck squamous carcinoma prognosis.	Non-coding RNA models need extensive validation	AI may provide new molecular targets for head and neck squamous carcinoma treatment. Exploring AI applications in epigenetic profiling of head and neck squamous carcinoma.

Diagnostic Decision Support

Diagnostic accuracy is a cornerstone of effective HNC's management, as misdiagnosis or delayed diagnosis can significantly worsen patient outcomes. Across SRs, CNN-based CDSS consistently demonstrated superior performance on diagnostic imaging tasks compared with conventional radiological interpretation. These systems were particularly effective in processing high-dimensional data from CT, MRI, PET, and ultrasound (US) imaging, identifying subtle abnormalities often overlooked by human observers [19,35].

A key strength of CNN-driven diagnostic CDSS lies in their ability to capture complex spatial hierarchies within imaging data. For instance, in thyroid cancer, US-based AI systems achieved pooled sensitivities above 90% and specificities of 85-90%, which, in some cases, surpassed the diagnostic performance of experienced radiologists [20,22,47]. Importantly, these tools also demonstrated utility in standardizing diagnostic workflows, potentially reducing inter-operator variability, a persistent problem in US interpretation.

Beyond imaging, CNN-based CDSS have shown promise in histopathological evaluation, where manual interpretation is subject to fatigue and intraobserver variability. Several reviews highlighted how CNN-driven histopathology models achieved high accuracy in differentiating malignant from benign oral lesions, supporting earlier detection and potentially reducing the need for invasive biopsies [21,17]. Integration of such systems into pathological workflows could accelerate turnaround times and enhance diagnostic confidence.

Collectively, these findings indicate that CNN-based CDSS not only enhance diagnostic precision but also serve as important adjuncts to clinicians, offering consistent and scalable support tools that could be particularly impactful in resource-limited healthcare settings.

Treatment Planning and Intraoperative Support

CNN-based CDSS also play an increasingly important role in therapeutic decision-making, particularly in radiation oncology and surgical planning. SRs consistently reported the potential of CNN-driven models to automate tumor segmentation and organ-at-risk delineation, two highly labor-intensive steps in radiotherapy planning. Compared to manual contouring, AI-supported delineation was faster, reduced variability between clinicians, and achieved accuracy levels close to expert consensus [6,49]. This is particularly relevant for HNC due to the anatomical complexity of the region and the need for precision to avoid damage to critical structures.

Another promising area is the use of AI-CDSS for predicting treatment-related toxicities, such as xerostomia or dysphagia, following radiotherapy. Reviews noted that radiomics-enhanced CNN models could identify patients at higher risk of adverse events before treatment, enabling clinicians to adapt dose plans accordingly [5,38]. By shifting toxicity prediction upstream in the treatment workflow, these systems align with the principles of preventive oncology and personalized care.

In surgical oncology, CDSS integrated with hyperspectral imaging (HSI) and optical coherence tomography were highlighted as valuable intraoperative tools. These AI-supported systems provided real-time tissue characterization, enabling surgeons to distinguish malignant from normal tissue during resection [23,41]. Such intraoperative decision support reduces the risk of positive margins, minimizes the need for re-operation, and improves functional and cosmetic outcomes.

Overall, CNN-based CDSS demonstrate strong potential to streamline therapeutic workflows, reduce human error, and extend support beyond pre-treatment planning into dynamic intraoperative environments.

Prognostic Decision Support and Risk Stratification

Prognostic modeling is another area where CNN-based CDSS have shown substantial promise. Accurate predictions of recurrence, metastasis, and survival are essential for guiding treatment intensity and follow-up strategies in HNC. SRs found that AI-driven prognostic models consistently outperformed conventional staging systems when multi-modal data were integrated [4,31].

CNN-based radiomics frameworks were particularly effective in extracting quantitative imaging biomarkers associated with disease progression. For instance, studies included in the reviews demonstrated that models trained on radiological features could predict locoregional recurrence and distant metastasis with higher predictive accuracy than standard clinical models [48,51]. These models not only stratify patients into high- and low-risk categories but also provide clinicians with actionable insights for tailoring follow-up intensity and adjuvant therapies.

Moreover, CNN-based prognostic CDSS extended beyond imaging, incorporating molecular and genomic data to refine risk prediction. By combining genomic alterations, protein expression patterns, and clinical data, these systems demonstrated the capacity to model tumor biology more holistically. This integration strengthens their relevance in precision oncology, where decisions must account for tumor heterogeneity.

Despite these advancements, reviews consistently cautioned that prognostic CDSS remain limited by small sample sizes, retrospective study designs, and a lack of external validation. Without rigorous validation in prospective cohorts, the clinical utility of these systems remains theoretical. Nonetheless, the promise of CNN-based prognostic models lies in their potential to enable personalized surveillance strategies, optimize resource allocation, and ultimately improve patient outcomes by identifying those most likely to benefit from intensified treatment or closer follow-up.

Methodological Strengths and Limitations of the Included SRs

The methodological quality of the included SRs varied. On the positive side, all studies employed multi-database searches, often including major medical and engineering databases such as PubMed, Scopus, IEEE Xplore, and Cochrane Library, enhancing the comprehensiveness of their evidence bases. Most reviews defined clear inclusion criteria centered on AI applications in HNC contexts and included a broad spectrum of ML and DL techniques.

Two reviews conducted MAs, enabling quantitative synthesis of diagnostic accuracy measures [20,22]. These efforts enabled the generation of pooled sensitivity, specificity, and diagnostic odds ratios (DORs), thereby strengthening the evidentiary base regarding AI’s diagnostic potential for thyroid nodules. Other reviews adopted thematic syntheses and performance summaries, which offered qualitative insights into trends, tools, and clinical use cases.

Nonetheless, several methodological limitations were noted. A key concern across reviews was heterogeneity, stemming from variability in AI model types, imaging modalities, datasets, and evaluation metrics. This heterogeneity impeded meta-analytic pooling in some cases and limited the generalizability of results. Studies by Kavyashree et al. and Wu et al. acknowledged challenges related to limited sample sizes, lack of external validation, and overfitting, which diminish the robustness of reported findings [21,23].

Among the SRs analyzed, the AMSTAR-2 checklist was explicitly used only in Mäkitie et al. and Sritharan et al. [1,3]. The Prediction Model Risk of Bias Assessment Tool (PROBAST) tool was applied by Adeoye et al. and mentioned by Araújo et al. to assess bias in prediction models [5,30]. Quality Assessment of Diagnostic Accuracy Studies-2 (QUADAS-2) was used for diagnostic accuracy evaluations in Li et al., Kim DH et al., and Kim JS et al. [18,43,44]. The Grading of Recommendations Assessment, Development and Evaluation (GRADE) approach was employed to assess the certainty of the evidence in Araújo et al. [5]. Moreover, aside from the few exceptions noted above, none of the remaining included reviews reported undertaking formal quality assessments using validated tools such as AMSTAR-2 or Risk of Bias in Systematic Reviews (ROBIS) [1,3,4,18,30,43,44]. The widespread absence of standardized risk-of-bias assessments raises significant concerns about the internal validity, methodological transparency, and reproducibility of these reviews. Without employing validated critical appraisal instruments, there is a greater risk that systematic errors, selective reporting, and methodological flaws may undermine the reliability of the evidence synthesis. Consequently, the findings and conclusions drawn by many of these reviews must be interpreted with caution, underscoring the urgent need for future SRs in this field to adopt rigorous quality assessment frameworks to strengthen the credibility of their contributions to clinical practice and research.

Additionally, reporting inconsistencies, such as missing performance metrics (e.g., precision, recall, area under the curve (AUC)), were observed across multiple studies, complicating cross-study comparisons. Several reviews also failed to address the interpretability of AI models, a critical factor in clinical adoption.

Publication bias was another issue, particularly in reviews that included studies sourced from grey literature and non-indexed platforms (e.g., Google Scholar). The use of such sources without rigorous filtering increased the likelihood of including lower-quality studies. Furthermore, some reviews did not clearly distinguish between retrospective and prospective validations, making it difficult to assess the findings' real-world applicability.

Discussion

This umbrella review demonstrates that AI, when applied through CDSS and particularly CNNs, offers meaningful contributions to the management of HNC. The review highlights how AI models are evolving from experimental tools into clinically relevant decision aids across diagnosis, treatment planning, and prognosis. These findings address a critical gap in the literature, where previous reviews often drew broad conclusions across disparate AI applications, potentially obscuring specific clinical implications.

Diagnostic Applications

Evidence consistently supports the diagnostic utility of CNN-based CDSS in both radiological and histopathological domains. As noted in prior reviews, CNNs can detect disease patterns with sensitivity and specificity comparable to those of expert clinicians [19,20,22,35,47]. The clinical relevance of these systems lies not only in their diagnostic accuracy but also in their ability to reduce variability among radiologists and pathologists. This is particularly significant for thyroid and oral cancers, where early and accurate detection substantially alters treatment trajectories [17,21]. Integration of diagnostic CDSS into routine workflows could therefore improve reliability, accelerate decision-making, and expand access to high-quality care in under-resourced settings.

Treatment Planning and Intraoperative Support

CNN-driven CDSS are increasingly integrated into therapeutic decision-making, particularly within radiotherapy planning. Reviews indicate that automated tumor delineation, dose prediction, and organ-at-risk identification achieved high levels of accuracy and reproducibility, alleviating a traditionally time-intensive process [6,49]. These advances align with precision oncology, where minimizing collateral tissue damage is crucial for functional preservation. Moreover, AI-assisted toxicity prediction models highlight the potential of CDSS to anticipate treatment complications such as xerostomia or dysphagia, offering clinicians the opportunity to adapt regimens before harm occurs [5,38].

In surgery, CNN-based intraoperative support tools integrating HSI and OCT extend decision-making into the operating room, providing surgeons with real-time guidance on tissue margins [23,41]. This capability underscores the breadth of CDSS applications, from pre-treatment planning to intraoperative navigation, and highlights their role in enhancing both efficiency and safety across the treatment continuum.

Prognostic Modeling and Risk Stratification

The prognostic potential of CNN-based CDSS was also evident across the included reviews. These models, leveraging radiomics and multi-modal data integration, consistently outperformed conventional staging systems in predicting recurrence, metastasis, and survival [4,31,48,51]. Importantly, prognostic CDSS not only stratify patients but also guide personalized surveillance strategies, ensuring that high-risk individuals receive closer follow-up and targeted interventions. The incorporation of molecular and genomic data further positions CNN-based models within the broader framework of precision oncology, where treatment decisions must account for tumor heterogeneity.

Nevertheless, most prognostic systems remain constrained by retrospective data, small cohort sizes, and the absence of external validation. Without prospective trials, their role in clinical practice is likely to remain supportive rather than definitive.

Methodological and Translational Considerations

The methodological quality of reviews addressing AI-CDSS varies. While some, particularly in thyroid cancer, offered meta-analytic evidence of diagnostic accuracy [20,22]; others relied heavily on narrative synthesis. Persistent challenges included model heterogeneity, dataset variability, a lack of standardized reporting metrics, and limited interpretability of CNNs. These limitations reflect a translational gap between promising research and clinical implementation.

A recurrent barrier is the “black-box” nature of CNNs, which may undermine clinician trust and adoption. Improving model explainability, alongside standardized evaluation protocols and robust external validation, will be essential for transitioning AI-CDSS into routine practice.

Implications for Future Research and Clinical Practice Challenges

The synthesis of evidence presented in this umbrella review demonstrates that AI-supported CDSS, particularly those leveraging CNN architectures, are poised to significantly influence the future of head and neck oncology. However, their successful translation into everyday clinical practice requires addressing several research, methodological, and implementation challenges.

First, prospective validation in large, multi-center clinical cohorts is essential. Much of the current evidence derives from retrospective analyses and single-institution datasets, which limit generalizability. Collaborative research efforts should prioritize data harmonization across centers and healthcare systems to enable the development of CDSS that perform consistently across diverse populations. Such initiatives will also help overcome issues of small sample sizes and limited demographic diversity that currently undermine external validity.

Second, there is an urgent need for standardized reporting and evaluation protocols for AI-based CDSS. Reviews repeatedly identified inconsistent use of performance metrics, with studies often emphasizing sensitivity or accuracy while neglecting other important measures such as recall, F1 score, or calibration. Establishing consensus guidelines for reporting AI outcomes in oncology would improve comparability between studies, facilitate MAs, and strengthen the reliability of synthesized evidence.

Third, model interpretability must be prioritized. CNN-based CDSS often function as “black boxes,” producing predictions without clear reasoning. This lack of transparency is a critical barrier to clinician trust and adoption. Emerging approaches such as saliency mapping, feature attribution, and explainable AI (XAI) techniques should be embedded in CDSS development to provide interpretable outputs that are easily understood by clinical end-users. Improved transparency will also facilitate regulatory approval and ethical oversight.

Fourth, integration into clinical workflows remains a practical challenge. While many models show promise in controlled research environments, few have been tested in real-world clinical settings where decision-making is complex and time-sensitive. Future research should move beyond algorithmic performance to evaluate how CDSS influence multi-disciplinary tumor boards, radiology-pathology consensus meetings, and surgical planning conferences. Understanding the human-machine interaction in these settings will be critical for effective implementation.

Fifth, ethical, legal, and data governance issues require sustained attention. The use of sensitive patient imaging, genomic, and clinical data necessitates rigorous frameworks for data privacy, secure storage, and responsible sharing. Moreover, clinicians and institutions must clearly define liability in cases where CDSS-influenced decisions lead to adverse outcomes. Establishing robust regulatory standards will be a prerequisite for safe clinical deployment.

Finally, from a clinical perspective, training and capacity building will be indispensable. As CDSS enters oncology practice, clinicians, radiologists, pathologists, and surgeons will need structured training in interpreting AI outputs and integrating them into patient management decisions. Such training should be multi-disciplinary and continuous, ensuring clinicians remain confident in using these evolving technologies.

## Conclusions

This umbrella review highlights the growing role of AI-supported CDSS, particularly those using CNN, in the management of HNC. Focusing specifically on this domain provides clearer insight into how AI-CDSS contributes across the cancer care continuum. The evidence suggests that CNN-based systems improve diagnostic accuracy in imaging and histopathology, support treatment planning through automated tumor delineation and toxicity prediction, and enhance prognostic assessment by enabling more precise risk stratification.

However, despite these promising developments, routine clinical implementation remains limited. Many studies report methodological constraints, including retrospective designs, small and heterogeneous datasets, insufficient external validation, and limited model interpretability, all of which restrict generalizability and reduce clinician confidence in AI-supported decision-making. Future research should therefore emphasize prospective multi-center validation, standardized reporting practices, and the development of transparent and interpretable AI models. Integrating CDSS into multi-disciplinary workflows while addressing ethical, legal, and governance considerations will be essential for translating technological advances into effective real-world oncology practice.
